# Bone Marrow Mesenchymal Stem Cell Senescence in the Development of Osteoporosis: Mechanisms, Interventions, and Future Directions

**DOI:** 10.2174/0115665240367456250323173450

**Published:** 2026-03-27

**Authors:** Chengen Li, Bo Li, Jiuchao Zhang, Kun Liu, Gang Du, Cunliang Guo, Zhenguo Yang

**Affiliations:** 1 Department of Orthopedia, Second Affiliated Hospital of Shandong University of Traditional Chinese Medicine, Jinan City, Shandong Province, 250000, China;; 2 Department of Integrative Chinese and Western Medicine, Tianjin University of Traditional Chinese Medicine, Jinghai District, Tianjin, 301617, China

**Keywords:** Bone marrow mesenchymal stem cells, osteoporosis, cell senescence, omega-3 fatty acids, cellular, molecular markers, SASP

## Abstract

Osteoporosis, a significant age-related disease, is marked by diminished bone density and an elevated risk of fractures, representing a considerable global health challenge. Bone marrow mesenchymal stem cells (BMSCs) are essential in maintaining bone integrity through their differentiation into osteoblasts, which are crucial for bone formation. Nevertheless, the aging of BMSCs diminishes their regenerative abilities and intensifies inflammation, thereby playing a critical role in osteoporosis pathogenesis. This review explores the intricate mechanisms of BMSC senescence and its influence on osteoporosis, detailing cellular and molecular markers, such as oxidative stress, the senescence-associated secretory phenotype (SASP), and pivotal signaling pathways, including P53, PI3K/mTOR, and autophagy. We assess current interventions aimed at reducing BMSC senescence, with an emphasis on pharmacological methods like melatonin and antioxidants, alongside non-pharmacological strategies, such as exercise and dietary supplementation with omega-3 fatty acids. Furthermore, the challenges and limitations of translating these strategies into clinical applications are addressed, highlighting the necessity for personalized medicine to accommodate treatment outcome variability. Future research directions should focus on emerging therapeutic targets and novel interventions, such as gene editing technologies and advanced tissue engineering techniques. By integrating these strategies, this review endeavors to enhance the understanding and treatment of osteoporosis, emphasizing the critical need to target BMSC senescence to develop effective therapies.

## 1 INTRODUCTION

Osteoporosis is a significant age-related condition characterized by weakened bones and an increased risk of fractures [1]. It predominantly affects postmeno-pausal women but also occurs in men, stemming from age-related changes in bone density and structure [1]. Globally, osteoporosis represents a major health risk, contributing to approximately 8.9 million fractures annually and significantly impacting health systems and individual lives [2].

BMSCs are crucial for maintaining bone health. They are essential for bone regeneration and emodeling, owing to their capacity to differentiate into osteoblasts, which are vital for new bone tissue for-mation [3]. Consequently, the health and functionality of BMSCs are directly linked to the overall quality and strength of bone. However, with aging, BMSCs undergo cellular changes that significantly impair their functionality, a process referred to as BMSC senescence. This senescence reduces their capacity to form bone, shifting the balance toward bone resorption over bone formation [4]. This shift is a primary contributor to the development of osteoporosis, particularly in older adults [5]. Recent studies have highlighted several advancements in understanding and potentially treating osteoporosis by targeting BMSCs. Research on insulin-like growth factor 1 (IGF-1) has demonstrated its ability to enhance BMSC proliferation and osteogenic differentiation, offering a potential therapeutic avenue to combat bone density loss [6]. Additionally, genetic screenings have identified specific markers that can predict an individual’s risk for osteoporosis, providing new methods to identify and potentially mitigate risk before disease progression [7]. Despite these advancements, the treatment and management of osteoporosis face significant challenges. Current interventions often do not reverse the condition but focus on slowing its progression and managing symptoms. This underscores the need for innovative approaches that address the underlying cellular changes, such as BMSC senescence intervention.

This review aimed to consolidate the current under-standing of BMSC senescence and its implications for osteoporosis, emphasizing recent research and emerging treatments. It also discussed the potential of targeting BMSC senescence as a novel therapeutic approach and future research directions that could lead to more effective management of osteoporosis and potentially other related conditions (Fig. **1**).

## 2 ROLE OF BMSC IN THE DEVELOPMENT OF OSTEOPOROSIS

BMSCs are multipotent stem cells residing within the bone marrow. They have the capacity to differen-tiate into various cell types, including osteoblasts, chondrocytes, and adipocytes [8]. This differentiation capability is essential for the repair and regeneration of bone tissue and for maintaining the bone marrow microenvironment. BMSCs are central to bone homeostasis, the dynamic process by which old bone is replaced with new bone tissue. They support bone remodeling by differentiating into osteoblasts that synthesize the bone matrix and promote mineralization, which is vital for maintaining bone strength and health [9].

The differentiation of BMSCs into osteoblasts is a highly regulated process involving multiple signaling pathways and molecular interactions. During osteoblast differentiation, BMSCs transition to pre-osteoblasts and subsequently mature into osteoblasts that produce bone matrix proteins, such as collagen [5]. This process is governed by factors, including BMPs (bone morphogenetic proteins) and WNT signaling, which are critical for activating key transcription factors like RUNX2, essential for initiating and progressing osteoblast differentiation [10]. IGF-1 also promotes osteogenic differentiation by enhancing BMSC proliferation and osteogenic capabilities [10, 11].

Conversely, the differentiation of BMSCs into adipocytes can be detrimental to bone health, especially when they predominate, a common occurrence in aging [12]. This adipogenic shift leads to decreased bone density, a key characteristic of osteoporosis. Recent studies have identified SIRT3 as a regulator in this process, where its overexpression is linked to increased adipogenic differentiation in aged BMSCs, suggesting a potential therapeutic target for mitigating age-related bone density loss [13]. Additionally, factors like PPARγ (peroxisome proliferator-activated receptor gamma) play significant roles in steering BMSCs toward adipocyte formation [14].

Dysregulation of BMSC function can result in improper bone formation and osteoporosis, notably through an imbalance favoring adipogenic differen-tiation over osteogenic differentiation, leading to increased bone marrow fat and reduced bone formation. This is particularly evident in age-related bone density loss, where enhanced adipogenic differentiation is observed due to changes in regulatory pathways, such as those influenced by SIRT3 [15]. Manipulating SIRT3 expression in BMSCs can shift their differentiation pathway, offering a potential target for therapeutic intervention in bone loss diseases [15]. Furthermore, oxidative stress and mitochondrial dysfunction in aged BMSCs significantly impair their osteogenic capacity. Addressing these mitochondrial issues could restore the osteogenic potential of aged BMSCs, thereby improving bone health and potentially reversing aspects of osteoporosis [16]. These insights underscore the pivotal role of BMSCs in bone health and the complex interplay of factors that regulate their function, emphasizing the importance of advanced therapeutic strategies in managing osteoporosis and other related bone diseases.

## 3 BMSC SENESCENCE

Cellular senescence is defined as the state in which cells cease to divide but do not die. This phenomenon plays a dual role: it prevents the proliferation of damaged, potentially cancerous cells, thereby inhibiting tumor growth, and contributes to aging and related diseases, such as osteoporosis, by accumulating senescent cells that lose their regenerative capacity [17]. Senescent cells exhibit distinctive phenotypic changes, including increased expression of senescence-associated β-galactosidase and secretion of pro-inflammatory cytokines, which affect tissue function and regeneration. The senescence of BMSCs is characterized by several cellular and molecular alterations, including decreased proliferation, increased senescence, and reduced differentiation potential towards osteoblasts, which are cells essential for bone formation [17]. As BMSCs age, their proliferative capacity diminishes, a change linked to shorter telomeres and decreased telomerase activity [18]. Aging BMSCs also show elevated expression of senescence markers, such as p16 and p21, which inhibit the cell cycle and promote senescence [19]. Additionally, there is a significant shift in their differentiation potential from osteoblasts to adipocytes, driven by changes in signaling pathways like WNT and BMP, which are crucial for osteoblast differentiation.

BMSC senescence is influenced by intrinsic factors, such as genetic instability, the DNA damage response, and oxidative stress, as well as extrinsic factors, including systemic inflammatory milieu and changes in the bone marrow microenvironment. These factors collectively contribute to the altered function of BMSCs in aging individuals. Research indicates that aging BMSCs have a slower proliferation rate due to increased cellular senescence and diminished responsiveness to growth factors [20]. The accumulation of senescent cells in the bone marrow impairs the regenerative capacity of the tissue and shifts their differentiation potential from predominantly osteogenic to more adipogenic lineage, negatively affecting bone health. This tendency for BMSCs to differentiate into adipocytes rather than osteoblasts is regulated by transcription factors, such as PPARγ and signaling pathways, including Wnt/β-catenin, and influenced by systemic regulators like glucocorticoids and estrogen [21]. For instance, studies have demonstrated that IGF-1 promotes BMSC proliferation and osteogenic differentiation through this pathway, offering a potential therapeutic target to mitigate bone density loss associated with aging [11].

Both systemic factors, such as hormones and cytokines, and local factors within the bone marrow niche, such as cellular interactions and extracellular matrix composition, influence BMSC function. Changes in extracellular matrix composition and cell-cell interactions within the bone marrow affect BMSC behavior. For example, glucocorticoids, acting through the glucocorticoid receptor, can impact the balance between osteoblastogenesis and adipogenesis of BMSCs, affecting bone remodeling and osteoporosis development [22]. Hormones, notably glucocorticoids and estrogen, profoundly affect BMSC function. The glucocorticoid receptor (GR) mediates these effects by altering gene expression patterns critical for BMSC differentiation [23]. Recent studies underscore the complex interplay of factors influencing BMSC function and senescence. For example, a study found that modulation of the Wnt/β-catenin pathway by IGF-1 can enhance both the proliferation and osteogenic differentiation of BMSCs, suggesting potential therapeutic avenues for osteoporosis treatment [24]. Another study highlighted the dose-dependent effects of glucocorticoids mediated through the GR, impacting BMSC differentiation and survival [22].

## 4 MECHANISMS INVOLVED IN BMSC SENESCENCE IN OSTEOPOROSIS

Recent studies have firmly established a link between the senescence of BMSCs and the development of osteoporosis. As BMSCs aging, their osteogenic potential diminishes while adipogenic activity increases, contributing to the deterioration of bone quality characteristic of osteoporosis. Clinical studies have reported a significant accumulation of senescent BMSCs in osteoporosis patients compared to healthy controls, underscoring the relationship between BMSC senescence and osteoporosis [25]. 

Understanding the molecular mechanisms of BMSC senescence is vital for developing effective therapies for osteoporosis. Key signaling pathways, including p53, Wnt/β-catenin, BMP, JAK2/STAT3, and calcium signaling, are critically involved in BMSC dysfunction during aging. Furthermore, non-coding RNAs, such as microRNAs (miRNAs) and long non-coding RNAs (lncRNAs), modulate BMSC senescence *via* pathways like SIRT1 and TRAF3 [26]. Epigenetic modifications, including DNA methylation, histone modification, and RNA modification, also play significant roles in driving BMSC senescence and the progression of osteoporosis [27]. Targeting these pathways and regulatory mechanisms may offer new therapeutic approaches to rejuvenate aged BMSCs and enhance bone regeneration (Fig. **2**).

 Liu *et al*. [28] used a mouse model to demonstrate that leucine-rich repeat-containing 17 (LRRC17) expression is significantly elevated in aged BMSCs, correlating with senescence. Knocking down LRRC17 in aged BMSCs reduced the expression of p16, p21, and p53, enhanced osteogenic differentiation, and improved mitochondrial function by promoting fusion and reducing oxidative stress. Mechanistically, LRRC17 regulates BMSC senescence by modulating mitochondrial dynamics and autophagy activity *via* the PI3K/mTOR pathway. Furthermore, the transplantation of LRRC17-knockdown aged BMSCs into ovariectomized mice significantly alleviated bone loss, increased bone mineral density (BMD), and improved bone microarchitecture, highlighting the therapeutic potential of targeting LRRC17.

In a separate study, Liu *et al*. [16] found that hydrogen sulfide (H2S) levels are inversely correlated with BMSC senescence. Treatment with an H2S donor, NaHS, reduced SA-β-gal, p16, and p21, enhanced proliferation, and osteogenic differentiation, and restored mitochondrial morphology in aged BMSCs. Mechanistically, H2S-mediated S-sulfhydration of SIRT3 rescued mitochondrial dysfunction by enhancing its interaction with the mitochondrial fusion protein OPA1 and reducing OPA1 acetylation. Additionally, SIRT3 S-sulfhydration maintained heterochromatin homeostasis by stabilizing interactions with Lamin B1 and KAP1 and preserving the expression of heterochromatin markers like H3K9me3. H2S-pretreated BMSCs significantly improved bone loss in ovariectomy-induced osteoporotic mice, demonstrating its therapeutic potential. However, further studies are needed to identify specific cysteine residues involved in SIRT3 S-sulfhydration and to explore the clinical applications of H2S.

Hu *et al*. [25] found that nucleosome assembly protein 1 like 2 (NAP1L2) expression is significantly elevated in senescent BMSCs, both *in vitro* and *in vivo*. Knockdown of NAP1L2 delayed senescence progression, while overexpression accelerated it. In an aging mouse model, elevated NAP1L2 expression correlated with increased bone marrow fat accumulation and decreased osteogenic capacity. Clinical data further revealed that NAP1L2 expression negatively correlates with BMD and CT values in patients with senile osteoporosis. Mechanistically, NAP1L2 regulates senescence through the NF-κB signaling pathway, modulating SASP factor expression and p53 signaling. Additionally, NAP1L2 suppresses osteogenesis by recruiting SIRT1 to deacetylate H3K14ac on promoters of osteogenic genes. Targeting NAP1L2 with anti-aging agents, such as nicotinamide mononucleotide (NMN), could mitigate senescence and enhance osteogenesis, offering a promising therapeutic strategy for age-related bone diseases [26-28].

Xiao *et al*. [29] reported that Y-box binding protein 1 (YBX1) expression decreased in BMSCs from aged mice and humans, correlating with increased senescence and impaired osteogenic differentiation. Conditional deletion of YBX1 in BMSCs accelerated bone loss and marrow fat accumulation, whereas overexpression of YBX1 promoted bone formation. They also found that Sciadopitysin treatment stabilized YBX1 by inhibiting its ubiquitin-mediated degradation, reduced mis-splicing of target genes, and mitigated age-related bone loss in aged mice. Mechanistically, YBX1 deficiency led to mis-splicing in genes critical for BMSC fate, including Fn1, Nrp2, Sirt2, Sp7, and Spp1, contributing to senescence and altered differentiation.

Li *et al*. [30] explored metabolic activity in aged BMSCs and its potential impact on senescence and regenerative capacity. They observed that aged BMSCs exhibited reduced proliferation, osteogenic and chondrogenic differentiation, and angiogenic function alongside higher adipogenic differentiation. Key regulators of mitochondrial biogenesis, such as PGC-1α, NRF1, and TFAM, and glycolytic enzymes, including HK1, PFK1, and PKM, were downregulated. Fatty acid binding protein 4 (FABP4) was identified as a key regulator of metabolic activity in aged BMSCs, with elevated expression correlating with reduced lipolysis and fatty acid oxidation. Treatment with CoQ10 enhanced osteogenic differentiation, reduced adipogenic differentiation, and improved mitochondrial function in aged BMSCs. These findings suggest that targeting FABP4 or related pathways and modulating mitochondrial function through agents like CoQ10 could improve the regenerative potential of aged BMSCs.

Optineurin (OPTN), a multifunctional protein involved in autophagy, vesicular transport, and ER stress, has been implicated in bone metabolism. OPTN promotes BMSC osteogenesis by inhibiting sene-scence and lipogenic differentiation. Mechanistically, OPTN regulates the expression of fatty acid-binding protein 3 (FABP3) through selective autophagy, delaying senescence and enhancing osteogenic differentiation [31]. These findings highlight OPTN as a potential therapeutic target for bone metabolic diseases.

Epigenetic modifications, including DNA methy-lation, histone modification, and RNA modification, play crucial roles in the aging of BMSCs. Changes in DNA methylation patterns, such as global hypomethylation and specific hypermethylation events, significantly influence gene expression and cellular function. Similarly, histone modifications, including methylation and acetylation, reshape chromatin structure and regulate gene accessibility. Targeting these modifications offers therapeutic potential; for instance, inhibitors of DNA methyltransferases (DNMTs), such as decitabine and azacitidine, can promote osteogenesis by reducing methylation at specific gene promoters. Likewise, inhibitors of histone deacetylases (HDACs) and histone methyltransferases, including EZH2 inhibitors, enhance osteogenic differentiation by modulating histone acetylation and methylation levels. Additionally, non-coding RNAs, such as miRNAs and lncRNAs, are key regulators of BMSC function. For example, miR-21 has been shown to promote osteogenesis, whereas lncRNA CIR inhibits chondrogenesis [3]. The mechanisms and roles of these epigenetic factors in BMSC aging and their contribution to the progression of osteoporosis have been systematically elucidated by Zhao *et al*. [30, 31].

In addition, Mei *et al*. [32] demonstrated that miR-203-3p is significantly upregulated in aged BMSCs, playing a critical role in promoting cellular senescence. It has been observed that the overexpression of miR-203-3p in young BMSCs induced senescence, decreased cell viability, and impaired osteogenic differentiation. Conversely, inhibition of miR-203-3p in aged BMSCs reversed these effects, restoring cell growth and reducing markers of senescence. Mechanistically, PBK was identified as a direct target of miR-203-3p, with miR-203-3p binding to the 3′-UTR of PBK mRNA, leading to its downregulation. Knockdown of PBK in young BMSCs mimicked the effects of miR-203-3p overexpression, promoting senescence and reducing proliferation, while PBK overexpression in aged BMSCs rescued these phenotypes. Further investigation revealed that PBK interacts with p53, promoting its ubiquitination and degradation, thereby inhibiting senescence. Notably, intrabone marrow injection of an adeno-associated virus carrying miR-203-3p inhibitors in aged mice improved bone density and trabecular parameters, highlighting the therapeutic potential of targeting miR-203-3p in osteoporosis.

Liang *et al*. [33] reported that diabetic BMSCs exhibit significant functional impairments, including reduced proliferation, migration, and increased senescence and apoptosis, compared to normal BMSCs. These changes were associated with a marked upregulation of miR-139-5p in diabetic BMSCs and human umbilical cord MSCs cultured under high-glucose conditions. Mechanistically, inhibition of miR-139-5p improved BMSC proliferation and migration while reducing senescence and apoptosis. Furthermore, overexpression of HOXA9 or c-Fos reversed the deleterious effects of miR-139-5p on BMSC function, suggesting that miR-139-5p plays a pivotal role in diabetic BMSC dysfunction. Supporting this, Long *et al*. demonstrated that miR-139-5p suppresses BMSC osteogenesis by inhibiting the Wnt/β-catenin signaling pathway, emphasizing its potential as a therapeutic target [34]. Wang *et al*. [35] identified TRAF3 as an important regulator of BMSC senescence, with its overexpression effectively inhibiting senescence markers. They further revealed that miR-363-3p regulates TRAF3 expression by directly targeting the TRAF3 3′-UTR, reducing TRAF3 mRNA and protein levels. Overexpression of miR-363-3p counteracted the inhibitory effects of TRAF3 on osteogenic and adipogenic differentiation while alleviating senescence, suggesting that the miR-363-3p-TRAF3 axis could serve as a novel therapeutic target for bone tissue engineering and osteoporosis treatment. In addition to miRNAs, Xiao *et al*. [36] systematically explored a wide array of differentially expressed small non-coding RNAs (sncRNAs) in BMSC senescence, including PIWI-interacting RNAs (piRNAs), small nucleolar RNAs (snoRNAs), small nuclear RNAs (snRNAs), and repeat-associated siRNAs (rasiRNAs). These findings expand the understanding of sncRNA-mediated regulatory networks in BMSC senescence and underscore their potential as therapeutic targets for addressing age-related bone disorders.

The N6-methyladenosine (m6A) modification, a reversible RNA modification, plays a critical role in various diseases, including osteoporosis. Huang *et al*. [37] reported that etoposide treatment significantly reduced the expression of Voltage-Dependent Anion Channel 3 (VDAC3) in BMSCs. Overexpression of VDAC3 decreased p21 and p16 and promoted osteogenic differentiation, as indicated by increased expression levels of RUNX2, COL1A1, and ALP. Furthermore, it was found that ALKBH5 overexpression inhibited the m6A modification of VDAC3, reducing its stability. Conversely, the knockdown of ALKBH5 suppressed cellular senescence and enhanced osteogenic differentiation, with these effects being reversed upon VDAC3 knockdown. Finally, YTHDF1 was identified as the m6A reader for VDAC3. Overexpression of YTHDF1 stabilized VDAC3 mRNA and enhanced the luciferase activity of the wild-type VDAC3 construct. These findings indicate that ALKBH5 promotes cellular senescence and inhibits osteogenic differentiation in osteoporosis by suppressing the m6A modification of VDAC3. This suggests that targeting the ALKBH5/VDAC3 axis may represent a promising therapeutic strategy for treating osteoporosis.

Estrogen deficiency is a major contributor to osteoporosis in postmenopausal women, resulting in increased bone loss and a heightened risk of fractures. This deficiency disrupts the balance between bone resorption and formation, and recent studies have emphasized the pivotal role of inflammation in this process. Inflammatory responses can damage the bone marrow microenvironment and induce cellular senescence, characterized by the secretion of pro-inflammatory cytokines and extracellular matrix-remodeling proteases, collectively known as SASP factors. These SASP factors exacerbate both senescence and inflammation, creating a self-perpetuating vicious cycle. Breaking this cycle is, therefore, a promising therapeutic approach for addressing postmenopausal osteoporosis. Wu *et al*. [38] reported that BMSCs derived from ovariectomized (OVX) mice exhibited a pronounced senescent phenotype with elevated SASP factor secretion. They also found that the JAK2/STAT3 signaling pathway was activated in OVX BMSCs, closely correlating with increased SASP factor expression. Estrogen treatment effectively mitigated senescence in OVX BMSCs by reducing SASP factor expression and restoring their osteogenic differentiation capacity. Similarly, ruxolitinib, a JAK inhibitor, significantly decreased SASP factor expression and senescence markers while enhancing osteogenic differentiation *in vitro*. Importantly, intraperitoneal administration of ruxolitinib in OVX mice prevented bone loss by reducing inflammation and senescence within the bone marrow. Micro-CT and histological analyses further confirmed the preservation of trabecular and cortical bone mass. These findings demonstrated that JAK inhibition not only alleviates senescence but also restores osteogenic function in OVX BMSCs, effectively preventing bone loss *in vivo*. Thus, targeting the JAK2/STAT3 pathway emerges as a promising therapeutic strategy for the treatment of postmenopausal osteoporosis.

Autophagy, a vital cellular process that maintains homeostasis by degrading and recycling cytosolic components, plays a significant role in regulating senescence and the secretion of SASP factors. Cellular senescence and autophagy share overlapping signaling pathways and effector mechanisms, with the tumor suppressor protein p53 serving as a key regulator of both processes. Zheng *et al*. [39] demonstrated that senescent BMSCs exhibit increased autophagy activity, as evidenced by enhanced monodansylcadaverine staining and elevated levels of autophagy-related proteins, including LC3 and Atg12. Lentivirus-mediated shRNA knockdown of p53 effectively reduced the levels of p16, p21, LC3, and Atg12. Furthermore, inhibition of autophagosome-lysosome fusion using Bafilomycin A1 (BaF1) confirmed increased autophagic flux in senescent BMSCs, which was attenuated following p53 knockdown. These findings highlight the therapeutic potential of targeting p53 to modulate autophagy and senescence in BMSCs. In a related study, Zheng *et al*. [40] reported that senescent BMSCs displayed enlarged morphology, elevated SA-β-gal activity (>80%), reduced BrdU incorporation (<20%), and upregulation of p16 and p21. They also identified increased levels of autophagy-related markers, such as ATG7, ATG5, and LC3-II/I, along with decreased levels of p62 in senescent BMSCs compared to early-passage cells. Importantly, inducing autophagy with rapamycin exacerbated the senescent phenotype while inhibiting autophagy with BaF1 alleviated senescence, as evidenced by reduced SA-β-gal staining and increased BrdU incorporation. Additionally, the levels of pro-inflammatory cytokines, IL-6 and IL-8, were elevated in senescent BMSCs, with rapamycin treatment further increasing their expression, while BaF1 treatment significantly reduced their levels. This study underscores the critical role of autophagy in regulating the expression of IL-6 and IL-8 during BMSC senescence, mediated by the transcription factor FoxO3a. These findings suggest that modulating autophagy and FoxO3a activity could represent viable strategies to mitigate BMSC senescence. Moreover, Chen *et al*. [41] explored the interplay between autophagy and ferroptosis in BMSC senescence. Their study revealed that ferroptosis-related genes (VEGFA, MAPK14, and SLC2A1) and autophagy-related genes (ATG9A and VEGFA) are pivotal in regulating both the senescence of BMSCs and their osteogenic differentiation. These insights further emphasize the intricate regulatory networks linking autophagy and ferroptosis to BMSC functionality, highlighting potential therapeutic targets for improving bone health in age-related conditions.

The Hedgehog signaling pathway, particularly Indian Hedgehog (IHH), has been identified as a critical regulator of aging and differentiation in BMSCs. Al-Azab *et al*. [42] recently demonstrated that IHH expression is significantly downregulated in senescent BMSCs. Knockdown of IHH resulted in increased SA-β-gal activity, upregulation of p53 and p16, and an enhanced SASP. Conversely, treatment with recom-binant IHH protein reduced SA-β-gal activity, corrected differentiation biases, and downregulated the expression of aging-related genes. Mechanistically, IHH regulates BMSC senescence by modulating the ROS/mTOR/4EBP1 signaling pathways. These findings highlight the importance of IHH in maintaining BMSC function and suggest that modulating this pathway could provide therapeutic benefits in combating cellular aging.

Xu *et al*. [43] reported that ubiquitin-specific protease 26 (USP26) expression decreases with age in BMSCs, correlating with elevated p16 and p21 and impaired self-renewal and multipotency. They further demonstrated that USP26-deficient BMSCs exhibited reduced proliferation and impaired osteogenic, adipo-genic, and chondrogenic differentiation capacities. The reintroduction of USP26 expression effectively reversed these age-related declines. Mechanistically, it was revealed that decreased expression of hypoxia-inducible factor-1α (HIF-1α) in aged BMSCs led to reduced USP26 transcription. HIF-1α binds to specific regions of the USP26 promoter, directly regulating its expression. This study demonstrated that USP26 combats age-associated declines in BMSC self-renewal and multipotency by maintaining mitochondrial homeostasis through the stabilization of SIRT2. Activation of the HIF-1α-USP26 pathway represents a promising therapeutic strategy to mitigate aging and enhance the therapeutic potential of BMSCs.

## 5 CURRENT INTERVENTION STRATEGIES FOR BMSC SENESCENCE

Effective interventions targeting the senescence of BMSCs are crucial for combating osteoporosis. These interventions encompass pharmacological and non-pharmacological approaches, as well as stem cell-based therapies. Moreover, strategies employed in treating other diseases, such as diabetes and cardiovascular diseases, show potential in addressing BMSC dysfunction and promoting bone health.

Senolytics, a class of drugs designed to selectively eliminate senescent cells, have emerged as a promising therapeutic option for age-related diseases, including osteoporosis [44]. Key senolytics include dasatinib, quercetin, and fisetin. Sun *et al*. [45] demonstrated that quercetin treatment significantly reduced the proportion of senescence-associated SA-β-gal-positive cells, decreased mRNA expression of senescence markers (p21) and SASP-associated genes (IL6, TNF-α, IL1α, and IL1β), and restored the colony formation ability of BMSCs. Furthermore, quercetin reversed increased DNA damage response markers (γ-H2AX) and decreased expression of epigenetic markers (H3K9me3, HP1α, and LAP2). Mechanistically, quercetin was found to inhibit the accumulation of cytoplasmic double-stranded RNA (dsRNA) and the expression of RIG-I and phosphorylated TBK1 (p-TBK1), key components of the RNA-sensing pathway. Additionally, the knockdown of RIG-I reduced the proportion of SA-β-gal-positive cells and reversed the senescent phenotype induced by H2O2, thereby improving osteogenic differentiation capacity in senescent BMSCs. Importantly, both quercetin treatment and RIG-I knockdown restored osteogenic differentiation potential in senescent BMSCs, highlighting their therapeutic potential. Moreover, the combination of dasatinib and quercetin has been shown to reduce the burden of senescent cells and alleviate physical dysfunction in aged mice. These findings underscore the potential of senolytic therapies to mitigate BMSC senescence and improve outcomes in osteoporosis.

Melatonin, a hormone primarily secreted by the pineal gland during the night, is widely recognized for its role in regulating sleep-wake cycles. In addition to its effects on sleep, melatonin possesses notable antioxidant, anti-inflammatory, and anti-aging properties, positioning it as a promising therapeutic agent for improving bone health, especially in aging populations. Xie *et al*. [27] observed that melatonin levels in both human bone marrow and mouse plasma decline with age. Specifically, melatonin levels were significantly lower in human donors over the age of 60 compared to those under 45, and similarly, aged mice displayed reduced melatonin levels compared to young adult mice. In this study, nuclear receptor binding SET domain protein 2 (NSD2), a histone methyltransferase responsible for H3K36me2 modification, was identified as a key gene downregulated in senescent BMSCs. They found that NSD2 expression was significantly lower in aged BMSCs compared to their younger counterparts, and this downregulation was associated with diminished osteogenic potential. Notably, melatonin treatment restored both NSD2 expression and H3K36me2 levels in senescent BMSCs. This restoration was linked to increased chromatin accessibility at the promoters of osteogenic genes, including RUNX2 and BGLAP, thereby promoting enhanced osteogenic differentiation. *In vitro* experiments further demonstrated that melatonin significantly improved the osteogenic potential of senescent BMSCs, as indicated by increased alkaline phosphatase (ALP) activity and mineralization. Importantly, this study also found a negative correlation between melatonin levels and the severity of senile osteoporosis in clinical samples. Moreover, in an aging mouse model, melatonin treatment improved bone microarchitecture and increased bone volume density. Collectively, the study concludes that melatonin enhances osteoblastogenesis in senescent BMSCs through NSD2-mediated chromatin remodeling.

Chen *et al*. [46] reported that BMSCs from high-fat diet (HFD)-fed mice exhibited increased markers of senescence, such as SA-β-gal, γH2A.X, and p21, alongside a decreased proliferative capacity. These senescent BMSCs also demonstrated impaired osteoblastic differentiation, characterized by reduced expression of key osteogenic genes. Further investigation using ATAC-seq revealed decreased chromatin accessibility at vitamin D receptor (VDR)-binding sites, suggesting that diminished VDR signaling plays a critical role in BMSC senescence. Treatment with a VDR activator, 1,25(OH)2D, in HFD-fed mice was found to reverse BMSC senescence, promote osteogenesis, and enhance bone mass. Mechanistically, VDR activation lowered intracellular reactive oxygen species (ROS) levels and preserved mitochondrial function in BMSCs. Additionally, the study identified superoxide dismutase 2 (SOD2) as a downstream target of VDR signaling. In HFD-fed mice, reduced SOD2 expression was linked to increased ROS levels and BMSC senescence. However, VDR activation restored SOD2 expression, alleviated ROS accumulation, and mitigated both BMSC senescence and bone loss induced by the high-fat diet. These findings suggest that VDR activation could serve as a potential therapeutic strategy for osteoporosis associated with high-fat diets.

Kynurenine (KYN), a tryptophan metabolite, has been shown to significantly elevate ROS levels in BMSCs, as evidenced by increased hydrogen peroxide production and heightened oxidative stress [47]. KYN also impairs mitochondrial respiration and energy production, which, in turn, leads to reduced osteogenic differentiation. Furthermore, KYN inhibits autophagy by decreasing LC3B-II and LAMP levels while promoting the accumulation of p62. Additionally, KYN induces early senescence in BMSCs, as indicated by increased SA-β-gal activity and upregulation of p21 expression, a process mediated through the p53/p21 signaling pathway. *In vivo*, KYN supplementation in mice resulted in decreased bone mass, reduced trabecular bone volume, and diminished osteoblast activity while simultaneously increasing osteoclast activity and marrow adiposity, features that closely resemble the aging phenotype. These findings highlight the detrimental effects of KYN on BMSC function and bone health.

Erxian Decoction (EXD) is a traditional Chinese medicine formula commonly used to treat menopausal syndromes. Recent studies have reported that EXD exerts a protective effect on aging BMSCs. Huang *et al*. [48] isolated exosomes from the serum of EXD-fed rats (EXD-Exos) and applied them to hydrogen peroxide (H₂O₂)-induced aging BMSCs. They found that EXD-Exos significantly mitigated H₂O₂-induced senescence in BMSCs, as evidenced by reduced expression of senescence-associated proteins (p21, p53, p16), decreased β-galactosidase staining, and restored cell cycle progression. Furthermore, EXD-Exos improved mitochondrial membrane potential, enhanced mitophagy, and reduced ROS levels in BMSCs. Notably, EXD-Exos preserved the osteogenic differentiation potential of BMSCs by upregulating Runx2 and OCN and downregulating PPARγ. These findings suggest that EXD and its derived exosomes offer a promising therapeutic strategy for age-related diseases, particularly osteoporosis.

Yi *et al*. [49] found that treatment with Desferal^®^ significantly reduced both SA-β-gal activity and ROS levels in senescent BMSCs, indicating a reduction in oxidative stress. Moreover, short-term treatment with Desferal^®^ rejuvenated senescent BMSCs by enhancing their proliferation, osteogenic differentiation potential, and overall stemness while simultaneously decreasing senescence markers. These results suggest that Desferal^®^ may effectively mitigate BMSC senescence and promote their regenerative capacity.

Cellular senescence is often accompanied by a decline in nicotinamide adenine dinucleotide (NAD+) levels. NAD+ is a vital coenzyme involved in cellular metabolism and DNA repair, and its reduction is closely associated with aging and cellular senescence. Wang *et al*. [50] reported that exogenous NAD+ treatment significantly decreased the number of SA-β-gal-positive cells and increased cell viability in senescent BMSCs. Additionally, exogenous NAD+ treatment reduced intracellular ROS levels in these cells. Mechanistically, NAD+ supplementation upregulated Sirt1 expression and downregulated PARP1 expression in senescent BMSCs, underscoring the potential of NAD+ as a promising anti-aging agent for BMSCs.

Diabetes mellitus (DM) is a metabolic disorder characterized by elevated blood glucose levels, which can lead to various bone complications, including an increased risk of fractures and osteoporosis. High-glucose conditions inhibit the proliferation, migration, and osteogenic differentiation of BMSCs while also accelerating their senescence due to increased production of ROS [51]. Furthermore, Chang *et al*. reported that high glucose induces premature senescence in BMSCs through the upregulation of autophagy, a process mediated by ROS31. Phytic acid (PA), an antioxidant known for its anti-diabetic, anti-inflammatory, and osteogenic properties, has been shown to significantly enhance BMSC proliferation, increase ALP activity, mineralization, and the expression of osteogenic markers (ALP, OPN, RUNX2, and COL-1) under high-glucose conditions. Additionally, PA reduced ROS production, SA-β-gal staining, and the expression of senescence markers, such as P21 and P53, indicating its anti-senescence effects [51]. Mechanistically, PA enhances osteogenic differentiation and inhibits cellular senescence in BMSCs under high-glucose conditions *via* the ERK signaling pathway. In a type 2 diabetes mellitus (T2DM) mouse model, PA-treated BMSCs promoted more new bone formation in extraction sockets. These findings suggest that PA holds potential as a therapeutic agent for promoting bone regeneration, particularly in the context of diabetes-related bone complications.

Cordycepin, a natural derivative of adenosine, has demonstrated anti-senescence and antioxidant pro-perties. Recent studies have highlighted the therapeutic potential of exosomes derived from dental pulp stem cells (DPSCs), which are capable of rejuvenating senescent cells and promoting tissue regeneration. Wang *et al*. [52] investigated the potential of cordycepin-loaded DPSC-derived exosomes (CY@D-exos) in rejuvenating senescent BMSCs. They found that CY@D-exos significantly reduced sene-scence markers (P16, P21, and SA-β-gal) and enhanced the proliferative and osteogenic differentiation capacity of aged BMSCs. This effect was mediated through the activation of the NRF2 pathway and the maintenance of heterochromatin stability, suggesting that CY@D-exos could serve as a novel therapeutic strategy for promoting bone repair in osteoporosis patients. Additionally, Zhai *et al*. [53] reported that cordycepin treatment reduced oxidative stress and cellular senescence in BMSCs exposed to reactive oxygen species (R/M) conditions. Cordycepin also promoted osteogenic differentiation while inhibiting adipogenic differentiation in BMSCs. Mechanistically, cordycepin was found to interact with the PI3K p110α subunit, specifically binding to Ser-774 and Ser-854, thereby inhibiting the PI3K/Akt/FOXO3 signaling pathway and reducing oxidative stress and senescence in BMSCs. Importantly, the use of exosomes modified with a BMSC-affinity peptide (E7) to deliver cordycepin enhanced delivery efficiency to BMSCs both *in vitro* and *in vivo*. This delivery system demonstrated superior therapeutic effects compared to free cordycepin, with significant improvements in bone microarchitecture and a reduction in bone loss. These findings underscore the potential of cordycepin-loaded exosomes as a promising approach for bone regeneration and the treatment of osteoporosis.

Liang *et al*. [54] reported that the traditional Chinese medicine *Liuwei Dihuang* pill significantly reduced the proportion of senescent BMSCs, improved cell viability, and decreased the expression of senescence-associated markers, such as p53 and p21. Moreover, the *Liuwei Dihuang* pill enhanced osteogenic differentiation, as evidenced by increased ALP activity and calcium deposition, while simultaneously inhibiting adipogenic differentiation by reducing lipid droplet formation in BMSCs. *In vivo*, treatment with the *Liuwei Dihuang* pill increased the number of osteoblasts and mineralized bone, reduced osteoclast activity, and mitigated bone marrow adiposity. Mechanistically, the *Liuwei Dihuang* pill upregulated the expression of Yes-associated protein (YAP) and autophagy-related proteins, including LC3 and Beclin1, while downregulating p62. These molecular changes delayed BMSC senescence and restored osteogenic potential. Collectively, these findings suggest that the *Liuwei Dihuang* pill may represent a promising therapeutic strategy for postmenopausal osteoporosis by targeting BMSCs and modulating autophagy.

Neuritin, also known as candidate plasticity gene 15 (CPG15), is a neurotrophic factor primarily recognized for its role in neuroplasticity, including the promotion of neural dendrite and axon growth and the mediation of synaptic circuit formation. Recent findings by Wang *et al*. revealed that neuritin significantly attenuates senescence in BMSCs while enhancing their migratory capacity [20]. These results also underscore the poten-tial of neuritin as a therapeutic agent for mitigating BMSC senescence and addressing osteoporosis.

Ionizing radiation (IR) is widely used in cancer therapy but is often accompanied by severe side effects, including bone loss and osteoporosis, primarily due to its adverse effects on bone cells. Wei *et al*. reported that IR induces cellular senescence in BMSCs and promotes osteoclastogenesis. Their study demonstrated that engineered cerium oxide nanozymes, with a higher fraction of Ce³⁺ surface sites, effectively mitigate IR-induced cellular senescence and osteoclastogenesis. These nanozymes achieved this by neutralizing ROS and reducing DNA damage while simultaneously promoting osteogenic differentiation and mineralization [55]. Furthermore, Bai *et al*. revealed that irradiation induces BMSC senescence through activation of the JAK1/STAT3 signaling pathway, leading to increased secretion of SASP factors [56]. These insights provide valuable directions for developing targeted interventions to counteract radiation-induced bone damage.

Recent studies have highlighted the significant effects of exercise and dietary interventions on reducing BMSC senescence. Regular physical activity, especially weight-bearing and resistance training exercises, has been shown to enhance BMSC proliferation and differentiation into osteoblasts [57]. This process is facilitated by the increased mechanical load and stress that stimulate osteogenic pathways, ultimately improving bone density and reducing the markers of cellular senescence [57]. In parallel, dietary interventions, particularly those rich in antioxidants and anti-inflammatory nutrients, support the maintenance and function of BMSCs. Diets high in calcium, vitamin D, and omega-3 fatty acids have been reported to reduce oxidative stress and inflammation, which are critical factors in the aging of BMSCs. For example, omega-3 fatty acids, commonly found in fish oil, have been shown to enhance the osteogenic potential of BMSCs by modulating the expression of genes involved in bone formation [58]. Integrating regular physical activity with a balanced, nutrient-rich diet pre-sents a powerful strategy to combat BMSC senescence and enhance bone health, offering a holistic approach to managing and potentially preventing age-related bone diseases like osteoporosis.

Currently, although notable progress has been achieved in addressing aging BMSCs through gene-editing technologies or by transplanting healthy donor-derived BMSCs or autologously cultured BMSCs, research on osteoporosis remains in its early stages [28, 59, 60]. Further studies are urgently needed to deepen our understanding and develop more effective therapeutic strategies. BMSC transplantation presents significant potential as a therapeutic strategy for osteoporosis. However, further research is needed to thoroughly investigate its efficacy and underlying mechanisms. Additionally, comprehensive clinical trials are essential to validate these approaches and optimize their application in clinical practice.

## 6 DISCUSSION

This review explores the mechanisms and interventions targeting the aging of BMSCs and their role in osteoporosis. BMSC senescence contributes to osteoporosis by reducing the regenerative potential of cells and increasing inflammation. Pharmacological interventions, such as senolytics, and antioxidants show promise in mitigating BMSC senescence and enhancing osteogenesis. Non-pharmacological approa-ches, including regular exercise and dietary supplements like omega-3 fatty acids, support BMSC health and function.

Current interventions targeting BMSC senescence face several translational challenges and limitations. One significant challenge is the variability in treatment outcomes due to the heterogeneous nature of BMSCs derived from different patients. This heterogeneity can be attributed to genetic, epigenetic, and environmental factors, which affect the cells' response to therapies. Another major limitation is the lack of robust and consistent biomarkers to assess BMSC senescence and therapeutic efficacy. This gap hampers the ability to standardize treatment protocols and predict patient outcomes accurately. Moreover, the high cost and complexity of advanced interventions, such as gene editing and stem cell-based therapies, limit their accessibility and widespread adoption in clinical practice. Personalized medicine approaches, which tailor treatments based on individual patient profiles, are needed to address these issues. However, implementing personalized therapies poses challenges in terms of developing precise diagnostic tools and managing the logistical and financial aspects of individualized treatments.

Future research directions in BMSC senescence and osteoporosis treatment are focused on identifying emerging therapeutic targets and novel intervention strategies. One promising area is the development of advanced gene editing technologies, such as CRISPR-Cas9, which can precisely modify genes associated with BMSC senescence and rejuvenation. These technologies hold the potential to correct genetic defects and enhance the regenerative capabilities of BMSCs, paving the way for more effective and personalized therapies. Advanced tissue engineering approaches, including the use of 3D bioprinting and scaffold-based techniques, are also being explored to create supportive environments for BMSC growth and differentiation. Additionally, the development of synthetic biomaterials that mimic the natural bone matrix can provide a conducive niche for BMSC activity and bone regeneration.

Integrating systemic approaches to address both BMSC senescence and osteoporosis is another key research focus. This includes the combination of phar-macological agents, lifestyle modifications, and regene-rative therapies to create comprehensive treatment protocols. For instance, combining antioxidants, senolytics, and physical exercise can synergistically reduce oxidative stress, enhance BMSC function, and promote bone health. Moreover, investigating the role of non-coding RNAs and epigenetic modifications in BMSC senescence could reveal new therapeutic targets and strategies to mitigate senescence and improve bone regeneration.

## CONCLUSION

Overall, the future of BMSC-based interventions in osteoporosis treatment looks promising, with ongoing research aimed at overcoming current limitations and developing innovative therapies that offer long-term benefits. Advances in personalized medicine, gene editing, and tissue engineering are expected to significantly enhance the effectiveness and accessibility of these treatments, ultimately improving the quality of life for patients with osteoporosis and other age-related bone disorders.

## Figures and Tables

**Fig. (1):**
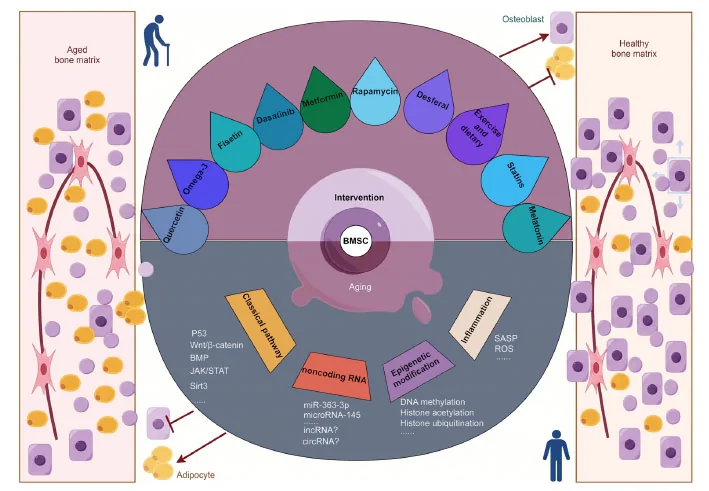
The role and intervention of BMSC senescence in the development of osteoporosis.

**Fig. (2):**
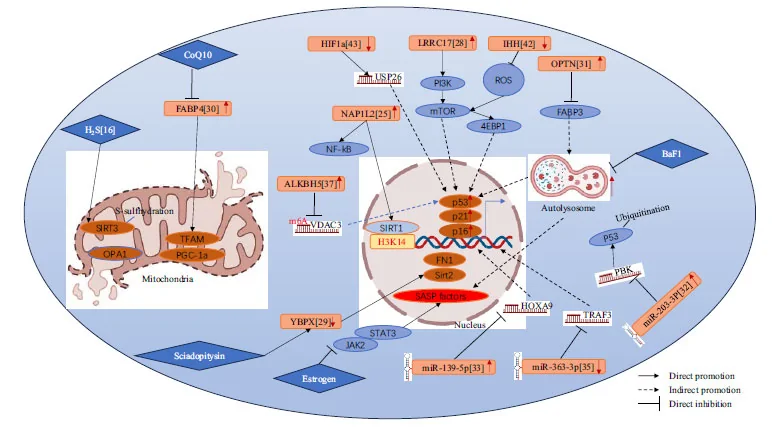
Mechanisms involved in BMSC senescence in osteoporosis. This diagram illustrates the complex signaling pathways involved in BMSC aging, encompassing regulatory mechanisms at the gene expression, post-transcriptional, and post-translational modification levels.

## LIST OF ABBREVIATIONS

BMSCs: Bone Marrow Mesenchymal Stem Cells
SASP: Senescence-associated Secretory Phenotype
IGF-1: Insulin-like growth Factor 1
